# Supplementary material for: A wheeze recognition algorithm for practical implementation in children
Source: PLoS One. 2020 Oct 8;15(10):e0240048. doi: 10.1371/journal.pone.0240048 (PMC7544038; doi:10.1371/journal.pone.0240048)
Supplement: Attachment — Submitted filename: Response to Reviewers.docx [file pone.0240048.s002.docx]

**Dear Dr. Daniel Dunea, Ph.D.
Academic Editor
PLOS ONE**

**Comments to the Author**

We have received the reviewers' comments. Based on their evaluation and my opinion after reading the revised manuscript, I recommend minor revision. Please address the comments raised by one of the reviewers and perform a quality control on the revised manuscript (potential typos, unclear statements, etc.) before resubmission. The scientific merit is more visible in the revised version, but few adjustments are required before publishing.

**Dear Dr. Daniel Dunea**

Thank you for sending the helpful comments from the reviewers on our manuscript. We have addressed all the comments and revised the manuscript as described below. We hope that you will find these revisions acceptable.

**Review 1 Comments to the Author**

The authors have responded appropriately to previous comments and the manuscript is much improved. There are a few additional changes that could improve the manuscript even further.

**Response to Referee 1**

Thank you for sending the helpful comments from the reviewers on our manuscript.

We have addressed all the comments and have revised the manuscript as described below. We hope that you will find these revisions acceptable.

In Table 1, please present age, mean (SD) overall, not for different age groups, as you have done with sex, n (%). Also present mean (SD) for height and weight.

**Reply 1**

We have added SD of age, height, and weight to Table 1. In addition, we have deleted the age range because we have already mentioned this in the text pertaining to different age groups. Furthermore, we have rewritten the range for height and weight as well as gender and added the percentage for different age groups. We found a few typographical errors in Table 1, which have now been corrected. This revision has not affected the results or conclusions.

**Table 1. Participant characteristics.**

| **Characteristic** | | **Value** |
| --- | --- | --- |
| **Age, mean (SD)** | | 57.5 months (43.1 months) |
|  | **2 months–11 months, n (%)** | 30 (14) |
|  | **12 months–23 months, n (%)** | 26 (12) |
|  | **24 months–35 months, n (%)** | 37 (17) |
|  | **36 months–47 months, n (%)** | 12 (6) |
|  | **48 months–59 months, n (%)** | 11 (5) |
|  | **60 months–71 months, n (%)** | 30 (14) |
|  | **72 months–83 months, n (%)** | 12 (6) |
|  | **84 months–95 months, n (%)** | 6 (3) |
|  | **96 months–107 months, n (%)** | 15 (7) |
|  | **108 months–119 months, n (%)** | 9 (4) |
|  | **120 months–131 months, n (%)** | 11 (5) |
|  | **132 months–143 months, n (%)** | 9 (4) |
|  | **144 months–155 months, n (%)** | 6 (3) |
|  | **Total, n (%)** | 214 (100) |
| **Gender, (Male:Female)** | | 138:76 |
| **Height, mean (SD), range** | | 101.7 cm (25.4 cm),  range: 56.7 cm to 160.5 cm |
| **Weight, mean (SD), range** | | 17.7 kg (9.3 kg),  range: 5.2 kg to 46.0 kg |

In Table 2, please remove the ± designation. You could present the characteristics as mean (SD) with the range below them e.g. 422 Hz (233 Hz) and then in the line below, range 100 Hz to 1380 Hz.

**Reply 2**

We have rewritten SD and range in Table 2.

**Table 2.** **Classification of sounds in all recorded sound files.**

| **1. Characteristics of wheeze sounds** | | |
| --- | --- | --- |
|  | Frequency, mean (SD), range | 422 Hz (233 Hz),  range: 100 Hz to 1380 Hz |
|  | Intensity, mean (SD), range | 18.9 dB (7.1 dB),  range: 3.0 dB to 44.0 dB |
|  | Duration, mean (SD), range | 388 ms (245 ms),  range: 100 ms to 1616 ms |
| **2. Type of wheeze sounds** | | |
|  | Monophonic wheeze, n (%) | 304 (37.4) |
|  | Polyphonic wheeze, n (%) | 509 (62.6) |
| **3. Noise** | | |
|  | Nasal congestion, n (%) | 115 (20.6) |
|  | Patient's crying or voice, n (%) | 138 (24.8) |
|  | Physician's voice, n (%) | 234 (42.0) |
|  | Ambient crying or voice, n (%) | 70 (12.6) |

**Reviewer #3:**

**Response to Referee 3**

Thank you for your helpful comments on our manuscript. We have addressed all the comments and revised the manuscript as described below. We hope that you will find these revisions acceptable.

Authors addressed all comments clearly. The revised version is well written and addressed all reviewers comments. Still it is not clear to me if two physicians lung sound interpretation was not match then how they solve it.

**Reply 3**

We have accordingly corrected this sentence in the revised manuscript (L107–108).

If the judgments of the two physicians did not match, the supervisor made the judgment [18].
